# Improved latrine coverage may reduce porcine cysticercosis: a comparative cross-sectional study, Busia County, Kenya 2021

**DOI:** 10.3389/fvets.2023.1155467

**Published:** 2023-07-05

**Authors:** Bernard Chege, Gideon Ndambuki, Maurice Owiny, Alice Kiyong’a, Eric M. Fèvre, Elizabeth A. J. Cook

**Affiliations:** ^1^Field Epidemiology and Laboratory Training Program (FELTP), Nairobi, Kenya; ^2^International Livestock Research Institute (ILRI), Nairobi, Kenya; ^3^Institute of Infection, Veterinary and Ecological Sciences, University of Liverpool, Liverpool, United Kingdom

**Keywords:** *Taenia solium*, porcine cysticercosis, prevalence, sanitation, public health, latrine access

## Abstract

**Introduction:**

Smallholder pig farming is an important economic activity for many poor, rural communities in developing countries. Porcine cysticercosis is a growing public health risk in countries where pig rearing is popular. A sanitation-based intervention to reduce the prevalence of open defecation was completed in Busia County, Kenya in 2016. We capitalized on this third party intervention to evaluate its impact on porcine cysticercosis prevalence.

**Methods:**

We conducted a comparative cross-sectional survey from August through to September 2021. Household selection was done using multistage sampling. Household questionnaire data on pig production, transmission, risk factors and awareness of porcine cysticercosis were collected from 251 households. Lingual palpation was used to test for cysticerci in 370 pigs while serum was tested for circulating antigen using Ag-ELISA. We compared results of our survey to an effective baseline, which was a near equivalent cross sectional survey conducted in 2012 before the third party sanitary intervention was established. The difference in prevalence was measured using Chi-square tests. Multivariable logistic regression analysis was used to identify risk factors for lingual cysts in pigs.

**Results:**

The prevalence of palpable lingual cysts was estimated to be 3.8% (95% CI 2.3–6.3%) (14/370). This was 6% (95% CI 0.8–13.9%; *p*-value 0.0178) lower than the prevalence reported in the pre-implementation period of 9.7% (95% CI: 4.5–17.6%). Circulating antigen was detected in 2 samples (0.54%, 95% CI: 0.2–1.9). Latrine coverage was 86% (95% CI: 81–90%), which was 11% (95% CI: 4.8–16.8%; *p* < 0.001) higher than the pre-implementation period coverage of 75% (95% CI: 71–79%). There was reduced prevalence of lingual cysts in pigs from households that had a latrine (OR = 0.14; 95% CI: 0.05–0.43; *p* < 0.001) and where pigs were confined or tethered (OR = 0.27; 95% CI: 0.07–1.02; *p* = 0.053).

**Conclusion:**

There was a reduction in the prevalence of porcine cysticercosis in Busia County over the study period from 2012 to 2021. This was not a trial design so we are unable to directly link the decline to a specific cause, but the data are consistent with previous research indicating that improved sanitation reduces porcine cysticercosis. Programs for controlling porcine cysticercosis should include a focus on sanitation in addition to other integrated One Health approaches.

## Introduction

Smallholder pig farming is an important economic activity for many poor, rural communities in developing countries due to the relative ease of pig rearing, low capital investment and the ability to utilize a variety of feeds including waste produce (1). Pigs multiply and grow quickly attaining market weight faster than other types of livestock (2).

Porcine cysticercosis is an infection of pigs by larval stages of *Taenia solium*, a zoonotic tapeworm transmitted among humans and between humans and pigs (3). Pigs are infected through ingestion of eggs shed in the feces of a human tapeworm carrier (4). Onchospheres hatch in the intestines from ingested eggs, migrate to striated muscles and develop into cysticerci. Upon ingestion of raw or undercooked pork containing cysticerci, human tapeworm infection occurs (5). The risk factors for porcine cysticercosis include poor sanitation (open defecation), the presence of human carriers for *T. solium*, undercooked pork, and free-roaming pigs (4).

Porcine cysticercosis has a worldwide distribution being most prevalent in Latin America, Asia and is considered endemic in pig keeping zones of the entire sub-Saharan region (6, 7). Porcine cysticercosis in endemic areas is of considerable medical and veterinary significance causing morbidity in humans and losses in animal production because of meat condemnation at slaughter (8). It is a growing public health concern in countries where pig rearing is becoming increasingly popular (9) and is considered the most important food-borne parasite globally (10).

In Kenya, *T. solium* cysticercosis is assumed to be rare since only a few cases in pigs are detected during routine meat inspection (11). However, studies conducted in western Kenya, particularly in Homabay, Siaya and Busia counties have found a high prevalence of porcine cysticercosis, reporting 34.4% prevalence for *Taenia* spp. cysticercosis in pigs by HP10 Ag-ELISA and 37.6% by lingual palpation in pigs as slaughter (12). Given the specificity of the antigen tests, these prevalence levels might vary but are an indication of the level of infection in this region (13). In Busia County, an extensive pig production system is predominantly characterized by free-ranging pigs (14). Pigs raised in free-range production systems may access human feces in areas where open defecation is a common practice due to lack of toilets. Consumption of pork meals with potentially infective porcine cysticerci has been reported in Busia County (15).

In the year 2012, the Ministry of Health initiated the national open defecation free (ODF) Kenya 2020 campaign framework, a nationwide community-led total sanitation (CTLS) campaign to end open defecation by 2020. The National Total Sanitation Program (NTSP) was implemented in Busia County with the aim to improve health outcomes through sanitation and hygiene promotion achieving open defecation-free status for the rural population in the entire Busia County (16). The project was dubbed Financial Inclusion Improves Sanitation and Health in Kenya (FINISH-INK) implemented by the African Medical and Research Foundation (AMREF) (16). Community members started building latrines, significantly changing the sanitation densities that existed before the intervention. The result of this intervention was the construction of 15,203 latrines increasing latrine coverage to 89% and the declaration of Busia County open defecation free in 2016 by the Ministry of Health, Kenya (16). Coverage of latrines improved significantly by 44% from 43 to 87% and open defecation reduced by 47% from 82 to 35% (17). A study of households conducted post-implementation of the project found a reduction of diarrhea cases reported by households from 48.3 to 10.6% over 5 years in Busia County (17).

Improved latrine coverage and usage at the community level is expected to facilitate proper disposal of human waste, reduce environmental contamination with *T. solium* eggs and pigs’ access to human feces, thus reducing transmission and prevalence of porcine cysticercosis. This study aimed to enumerate the effect of the sanitation intervention conducted in Busia County on the prevalence of porcine cysticercosis.

## Materials and methods

### Study site

Busia County was selected purposively because of its known popularity of pig rearing. Busia County is located in the western region of Kenya and has a population of 893,681 people with 198,152 households (18). It is comprised of seven sub-counties (Teso North, Teso South, Nambale, Matayos, Butula, Samia and Bunyala), 35 wards and 1,731 villages.

The Busia County pig population is 57,004 against the national population of 442,761 (18). Smallholder pig farming is practiced mainly under free-range or tethering, popular mainly due to low inputs, small space requirements and significant contribution to the local economy as a quick source of income. Pork is an affordable protein source, popular in the rural communities of western Kenya (14). However, consumption of poorly cooked pork is common (15).

### Study design (pre- and post-intervention)

This study was conducted using two study approaches combined into a comparative cross-sectional design. The first study involved a review of data generated during a cross sectional survey conducted between 2010 and 2012, referred to as the NTSP pre-implementation period (19, 20). The second was a cross-sectional study design during the NTSP post-implementation period (2021). A combination of these two study designs enabled us to compare changes in sanitation levels and porcine cysticercosis prevalence in the same area before and after NTSP implementation.

### Sample size determination

The sample size was calculated to detect a difference in the prevalence of porcine cysticercosis before and after improved sanitation (21). Since the prevalence of porcine cysticercosis by lingual palpation was 9.7% in the previous study of 93 pigs (20), we estimated that 190 pigs would be required in the repeated sample to show a statistically significant difference if the prevalence in the repeated sample was 4%. A design effect of 1.9 was applied due to the clustered nature of the sampling (22). The number of pig samples per cluster was 10. The adjusted sample size was 361 pigs.

### Sampling procedure

Sampling for the NTSP post implementation survey was carried out from August through to September 2021 where a multi-stage random sampling procedure was used to identify households. All seven (7) sub-counties were included to represent geographical distribution. The population of pigs by sub-county was established and the sample size for pigs was proportionally distributed.

In the first stage, pig-rearing villages in each sub-county were identified from a list of all villages of Busia County. Computer generated random numbers were used to select a total of 37 villages from the list of pig rearing villages. In the second stage, a list of pig rearing households in each village was generated by village elders (*liguluu* or *ejakait*) and computer generated-random numbers used to selected households.

A minimum of one (1) and a maximum of two (2) pigs were randomly selected proportional to herd size in each household. Pigs in the household that met inclusion criteria were assigned numbers, this was written on a piece of paper and folded. One (1) or two (2) folded papers were picked randomly dependent on the herd size.

### Exclusion and inclusion criteria

The inclusion criteria for data collection for the post-implementation of NTSP study were pig-keeping households. Inclusion criteria for the pigs were pigs without symptoms of ill health, more than 2 months of age and neither pregnant nor suckling sows. The exclusion criteria for the household survey were those households whose respondents could not provide required information due to inability to communicate, missed scheduled appointments and those that were selected for questionnaire pretesting. Exclusion criteria for pigs were those that were extremely emaciated, or could not be restrained for collection of a blood sample due to aggression.

### Sample collection (pre- and post-intervention)

Data used to determine the prevalence of porcine cysticercosis before implementation of the NTSP, were obtained from datasets generated during a cross-sectional survey conducted in Busia County in August 2010–July 2012 (19). During this study, the prevalence of porcine cysticercosis by lingual examination and HP10 Ag-ELISA was determined. Sensitivity for lingual palpation was estimated to be 16.1% (95% confidence interval (CI) 5–34%) and specificity 100% (97.5% one-sided CI 90–100%) (23). The sensitivity and specificity of the HP10 Ag ELISA are reported to be 89.5% (95% CI: 82.3–94.2) and 74% (95% CI: 56.6–87.6) (24). The HP10 Ag ELISA uses a monoclonal antibody to detect a high molecular weight metacestode glycoprotein (25). The variables considered from household data included: water sources, latrine availability, latrine use, latrine type, pig scavenging at latrines, and pig’s husbandry while those from pig’s data included: breed, sex, age, lingual examination results, and Ag-ELISA results.

Data for NTSP post-implementation period were primary sources including face-to-face interviews using a questionnaire and examination of pigs. During NTSP post-implementation, a structured electronic questionnaire developed in Epi Info^™^ (Centers for Disease Control and Prevention, Atlanta, United States) was administered to pig farmers. The household questionnaire was pre-tested by administering it to seven pig farmers in Matayos South, Busibwabo wards of Matayos Sub County. Data collected during the pretest was not included in the final analysis. The data collected during the household survey included respondent’s demographic information (gender, age, sub-county, ward, village, marital status, and religion), social-economic and sanitation data (source of income, availability and type of a latrine, hand-washing facilities). Additional data included pork consumption practices and knowledge (source of pork, method of cooking, method of rearing pigs, and knowledge of cysts in pork).

The pig selected for the study was restrained by inserting the wire loop end of a pig snare in the mouth and over the upper jaw and snout of the pig. The snare handle was held vertically in the other hand, put behind the upper canine teeth and pulled back to hold tight to the upper jaw stabilizing the head to facilitate insertion of a wooden stick horizontally into the mouth across the jaws to gag the mouth keeping it open. The tongue was pulled out rostrally holding it with a piece of cotton gauze to reduce slipperiness exposing its ventral surface which was observed and palpated for the presence of cysticerci. A cyst was defined as any small palpable whitish sac-like vesicle, approximately 1–2 mm in diameter on the ventral side of the tongue (26). Pigs with visible or palpable cysts on lingual examination were considered positive for cysticercosis.

Upon examination of pigs for cysts, we obtained 8 mL of a blood sample from the anterior vena cava using 20 g needles into plain vacutainer tubes which were then packed in ice, transported to the laboratory and centrifuged at 3,000 rpm for 25 min to obtain serum. Serum was then dispensed into 2 mL cryovials in aliquots, labeled with a unique identifier, barcoded and stored at −20°C. Laboratory sample collection and submission forms were filled indicating sample unique identifier, pig owner residence, date of sample collection, pig information (sex, age, breed), sample type, tests to be done and lingual examination results.

### Enzyme-linked immunosorbent assay

Laboratory diagnosis of porcine cysticercosis by detection of circulating cysticerci antigens was done at the International Livestock Research Institute (ILRI) laboratories in Nairobi using apDia cysticercosis Antigen (Ag) ELISA (apDia, Turnhout, Belgium). The sensitivity and specificity of the apDia ELISA are reported to be 82.7 and 86.3%, respectively (27). The apDia Ag ELISA uses different monoclonal antibodies (B158/B60) to detect antigen than the HP 10 Ag ELISA (28, 29). Since the results are not directly comparable true prevalence estimates were calculated as described below. Samples with detectable porcine cysticerci antigens were considered positive for porcine cysticercosis.

Laboratory procedures for the diagnosis involved adding samples to antibody-coated wells followed by incubation to allow antigens from viable cysticerci to bind to antibodies. Unbound serum proteins were then removed by washing and antigen-antibody complex in each well detected with specific peroxidase-conjugated B60H8A4 monoclonal antibodies. After removal of the unbound conjugate, the strips were incubated with a chromogen solution containing tetramethylbenzidine and hydrogen peroxide: a blue color developed in proportion to the amount of immune complex bound to the wells of the strips. The enzymatic reaction was stopped by the addition of 0.5 M H₂SO₄ and the absorbance values were determined at 450 nm and 630 nm using a microplate reader. The cut-off and antigen index for samples were calculated where a positive reaction corresponded to an antigen index above or equal to 1.3. An antigen index below or equal to 0.8 corresponded to a negative reaction while an antigen index between 0.8 and 1.3 corresponded to a doubtful result which was considered negative for the purpose of analysis.

### Data and statistical analysis

Pre-implementation period data from pigs was used to determine the prevalence of porcine cysticercosis pre- NTSP by lingual palpation and Ag-ELISA while household data was used to determine latrine coverage, use, type, and pig scavenging at latrines. Post-implementation data from household survey were combined with pig’s data in MS Excel (Microsoft Corporation) spreadsheet cleaned and exported to Epi Info^™^ (Centers for Disease Control and Prevention, Atlanta, United States) software for statistical analysis. Descriptive statistics for frequency and proportions were used to determine the magnitude of variables among respondents.

Post-implementation pig-level prevalence was calculated as the total number of pigs testing positive for cysticercosis either by lingual palpation or apDia Ag-ELISA divided by the total number of pigs examined. True prevalence accounting of the sensitivity and specificity of the tests was calculated using a Bayesian estimation implemented using the *truePrev* function in the prevalence package (30) of R.^1^

Change in prevalence of porcine cysticercosis and latrine coverage at pre-and post-NTSP implementation was used to evaluate the effect of improved sanitation on the prevalence. The existence of statistical difference in the prevalence of porcine cysticercosis, latrine coverage and latrine use at NTSP pre-and post-implementation was determined using Chi-square test at 95% confidence level and *p*-value < 0.05.

Independent variables were explored for association with porcine cysticercosis (lingual cysts) using multi-level logistic regression models developed using the *lme4* package in R statistical software (version 4.2.2) (31). The strength of association was determined using odds ratio (OR). Village was included as a random effect to account for clustering in the model. The factors that were considered in the analysis were presence of latrine, pork consumption, tapeworm shedding history, deworming history, epilepsy history, tethering pigs, and pig factors (breed, sex, age). A backward elimination approach was used to develop a multivariable model, including variables with *p-*value ≤ 0.1 on univariable analysis, and sequentially removing variables with the highest *p*-values to identify the best model. The *simulateResiduals* function from the *DHARMa* package was used to assess the validity of the final model by comparing the residuals plot vs. fitted values plot for each fixed effect for absence of clustering patterns or outliers, and that deviations from the empirical and expected quantile distribution were not significant (*p-*value > 0.05) (32).

### Ethical consideration

Approval to conduct this study was granted by the Institutional Research and Ethics Committee (IREC) of Moi University on March 25, 2021 Ref: IREC/2021/07, Approval No. 0003831 and the International Livestock Research Institute Institutional Animal Care and Use Committee (ILRI IACUC2021-16). The research license was obtained from the National Commission for Science, Technology & Innovation (NACOSTI), License No: NACOSTI/P/21/11274 and Ref No:772088.

Before traveling to the field for data collection, a formal communication was done to the Director of Veterinary Services, Busia County who provided written permission to conduct the study, Ref: CDVS/BSA/ RESEARCH/VOL/1/78.

Written consent was sought from eligible study participants in the selected households after introducing the study. Confidentiality of information obtained during the household survey and laboratory analysis of the sample was maintained throughout the process. This is by use of barcodes and password-protected computers and databases.

## Results

### Socio-demographic characteristics

A total of 251 respondents were interviewed during the household survey out of which 68.9% (173/251) were female (Table 1) and the mean age was 41.9 years (Range = 18–82). Respondents without formal education were 17.9% (45/251). The mean number of pigs per household was 3.4 (SD ± 5.4), with the majority of farmers 85.3% (214/251) keeping less than 4 pigs. The selected households kept other domestic animals including poultry 89% (224/251), cattle 75% (187/251), dogs 47% (118/251), goats 33% (83/251), and sheep 16% (40/251). The majority 40.6% (102/251) of the respondents depended entirely on animal production as their main source of income, 35.5% (89/251) (Table 1).

**Table 1:** Socio-demographic characteristics of participants, knowledge of cysticercosis and pig rearing (*n* = 251).

Key variable	Frequency	Percentage (%)
**Gender**		
Female	173	68.9
Male	78	31.1
**Education**		
Primary	146	58.2
Secondary	48	19.1
None	45	17.9
Tertiary	12	4.8
**Marital status**		
Married	206	82.1
Widow	27	10.8
Single	17	6.8
Separated	1	0.4
**Main source of income**		
Animal husbandry	102	40.6
Crop farming	89	35.5
Businesses	52	20.7
Casual labor	4	1.6
Formal employment	4	1.6
Knowledge on porcine cysticercosis*		
Identification of pork cysts	72	28.7
Know source of pork cysts	44	17.5
Having seen tapeworm proglottids	102	40.6
Households with epilepsy history	45	17.9
Do not know tapeworm transmission	249	99.2
**Pig rearing method**		
Tethering	114	45.4
Free-range and tethering	112	44.6
Total confinement	14	5.6
Free-range	11	4.4

*Multiple choice question.

### Pig husbandry practices

A total of 370 pigs were sampled. The pig breeds included exotic 1% (3/370), indigenous 68% (252/370), and crossbreeds 31% (115/370). Female pigs were most predominant at 54.6% (202/370) while the mean age of all pigs sampled was 6.9 months (SD ± 13.9), female pigs 7.4 months (SD ± 5.7) and male pigs 6.2 months (SD ± 4.7). There was a statistically significant difference between the mean ages of pigs by sex on the *t*-test (*p* = 0.03).

**Figure 1:**
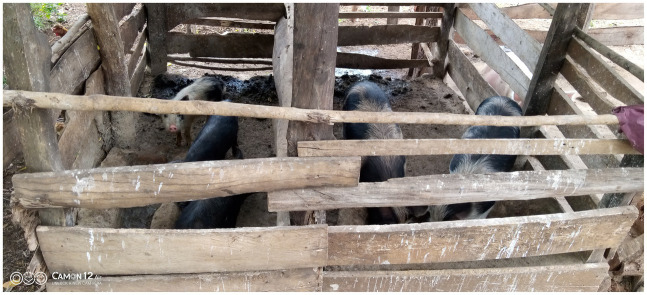
Pigs confined in a pen at Dip village, Teso North Subcounty, Busia County.

**Figure 2:**
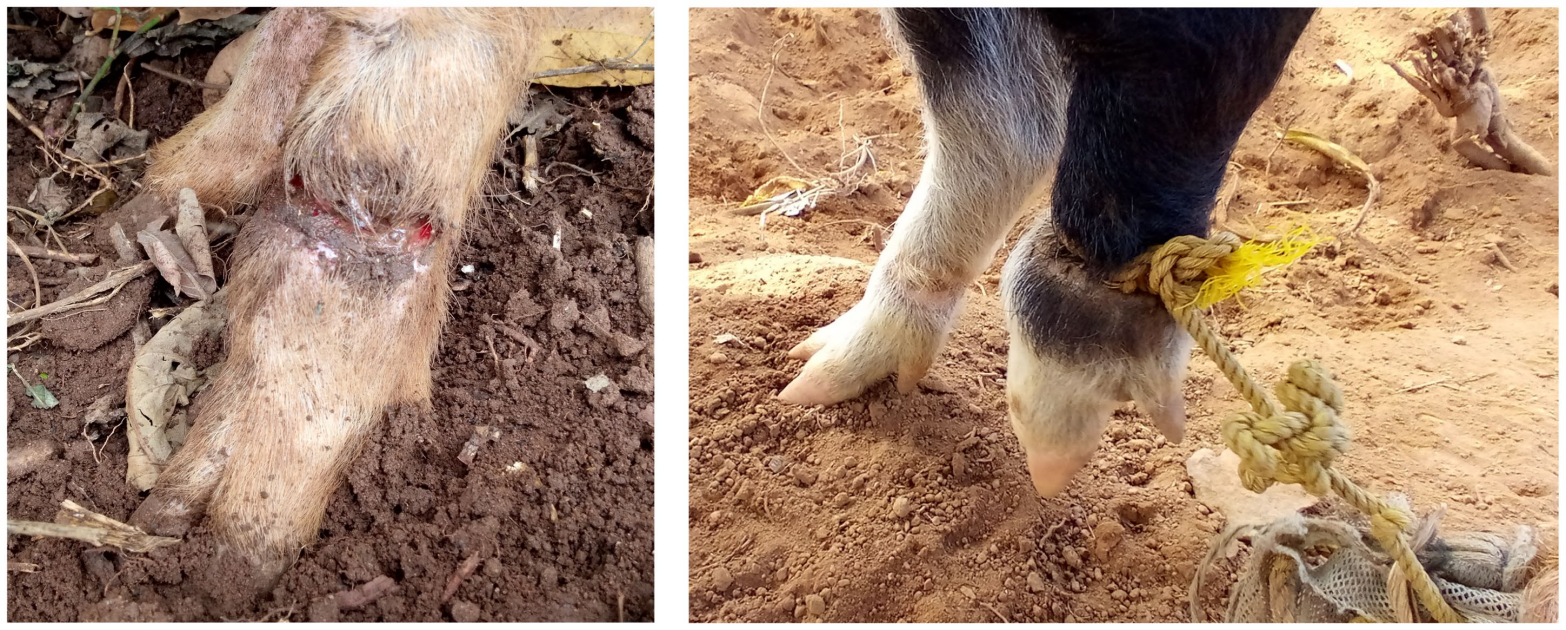
(Left) Septic wound on a pig leg caused by tether rope (arrow). (Right) Wounds caused by nylon tether rope.

Tethering was the most preferred method of rearing pigs, practiced by 45.4% (114/251) with a combination of both free-range and tethering also being practiced by 44.6% (112/251) (Table 1). Total confinement of pigs in pens (Figure 1) was practiced by 5.6% (14/251) while complete free range were 4.4% (11/251) (Table 1). Pigs were tethered by tying one end of a sisal or nylon rope around the lower limb above the dewclaws, while the other end was tied to a peg sunk into the ground or a shrub (Figure 2). Tethering can result in wounds on the pigs’ limbs that became septic when left unattended (Figure 2).

The main feed for pigs in Busia County is natural pasture fed during the day and supplemented with other feed types usually a mixture of ingredients in the evening. Leftover food is the main ingredient to supplement pig feed for the majority of households 78.5% (197/251). Alternative supplemental pig feed includes: uncooked fruit and vegetables 6.4% (16/251), commercial feed 6% (15/251), *ugali* (cooked maize flour meal) 6% (15/251), kitchen waste 2% (5/251) and uncooked animal products 1.2% (3/251). Other types of feeds given to pigs rarely or in small quantities include brewers’ mash, cassava roots, *omena* (Silver Cyprinid), rice bran, soya, fish by-products, and sweet potato tubers.

### Sanitation

The residents of Busia County obtained water for domestic use from various sources mainly wells 59% (148/251), river 14% (34/251), boreholes 9% (22/251), springs 8% (20/251), tap water 6% (15/251), and dam/lake 5% (12/251). During the cross-sectional study conducted in 2012 (NTSP pre-implementation period) the households having access to water from closed water sources were 56% (233/416) while during the post-implementation survey it was 70% (176/251) with a statistically significant difference in access to closed water sources between the two periods under observation (*χ*^2^ = 12.91, *p* < 0.001).

**Figure 3:**
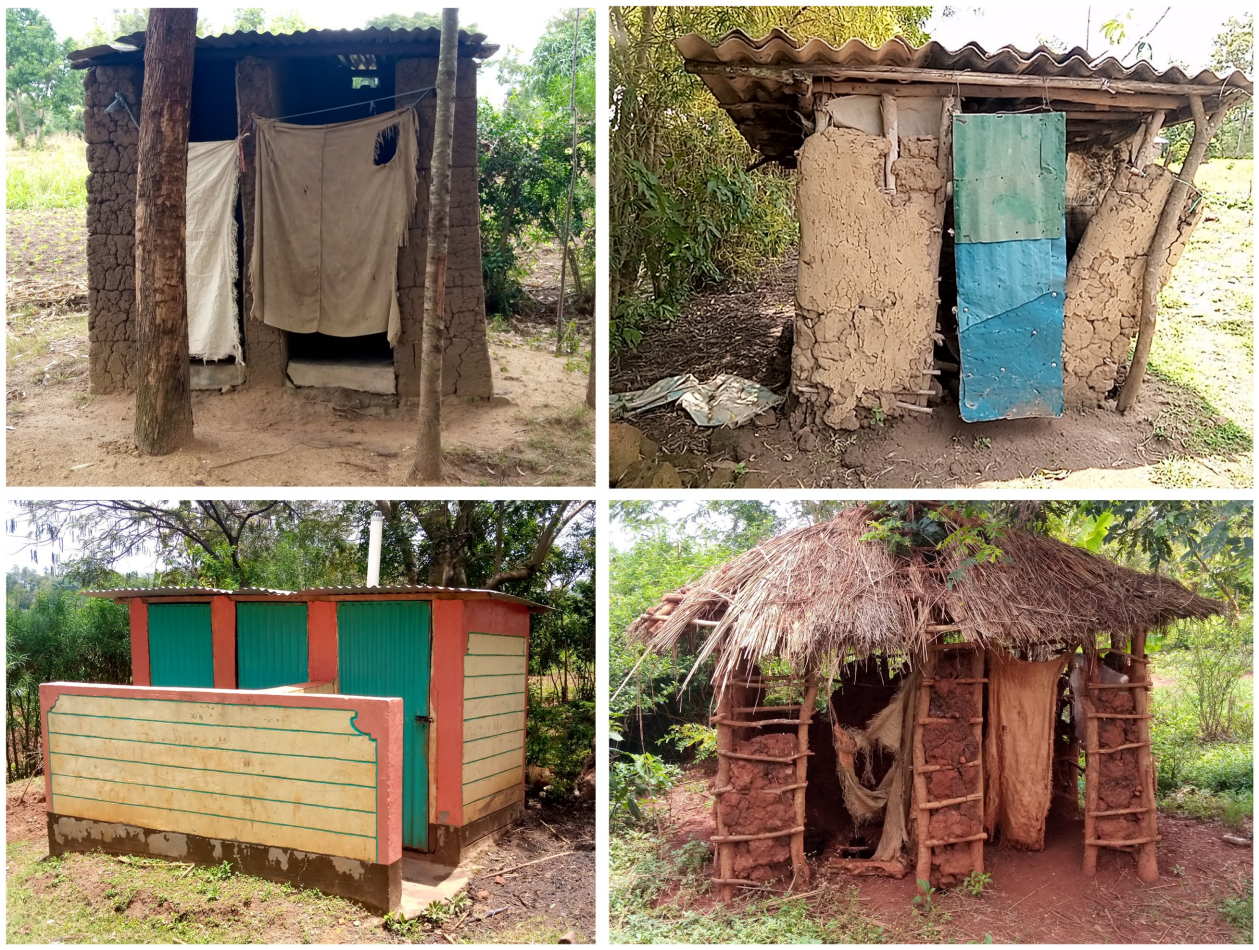
Types of latrines found among households visited, Busia County, Kenya, 2021.

During the NTSP pre-implementation period in 2012, latrine coverage was 75% (312/416) while latrine use was at 72% (301/416). Out of 312 households surveyed with a latrine in their compound, 29.2% (91/312) were completely closed, 6.4% (20/312) were open pit and 64.4% (201/312) were partially closed (Figure 3). Pigs scavenging around the latrines was at 5.1% (16/312).

During the NTSP post-implementation period, latrine coverage was 86% (215/251) (Table 2) while the use of latrines for disposal of human waste was at 97% (243/251). On direct observation, we found that all the latrines were being used as evidenced by a beaten path leading to the latrine, matting of human waste around the pit hole and signs of regular cleaning and 63% were closed. Latrine sharing was done by 26.3% (66/215), presence of handwashing facilities 25.5% (64/215) and pigs scavenging around the latrines was observed at 16.3% (41/215) of households.

**Table 2:** Comparison of latrine sanitation levels and porcine cysticercosis prevalence pre and post-implementation of NTSP.

Key variable	Pre-implementation	Post-implementation	Difference	95% CI	*p*-value
Latrine coverage	75% (312/416)	86% (215/251)	11%	4.8–16.8	<0.001
Latrine use	72% (301/416)	97% (243/251)	25%	20–30	<0.001
Lockable latrines	29% (91/312)	63% (135/215)	34%	25.5–41.8	<0.001
Scavenging at latrines	5% (16/312)	16% (41/215)	11%	5.8–16.8	<0.001
Lingual prevalence	9.7% (9/93)	3.8% (14/370)	5.9%	0.8–13.8	0.020
Ag-ELISA prevalence	17.2% (16/93)	0.54% (2/370)	16.7%	10.2–25.6	<0.001

Pre-implementation data (20).

There was a statistically significant difference in latrine coverage between the two periods with increased latrine coverage in the post implementation period (*χ*^2^ = 11.46, *p* < 0.001), increased latrine use (*χ*^2^ = 64.5, *p* < 0.001), and completely closed latrines (*χ*^2^ = 58.5, *p* < 0.001). However, pigs scavenging around the latrines increased in the post implementation period (*χ*^2^ = 18.2, *p* = <0.001).

### Knowledge on porcine cysticercosis, pork consumption, and parasite control

The respondents who observed cysts in pork were 28.7% (72/251) while 17.5% (44/251) indicated that they know the source of porcine cysts. However, amongst those who claimed to know the source of porcine cysts only 4.5% (2/44) could associate porcine cysts with pigs’ eating human feces. A total of 40.6% (102/251) respondents had been seen tapeworm proglottids in human stool while 17.9% (45/251) reported household members with a history of epilepsy. The majority of the respondents (99.2%; 249/251) did not know the route of transmission of the tapeworms.

The majority 89% (223/251) of the respondents interviewed consumed pork with 25.1% (56/223) consuming it often (once in a week); 39.5% (88/223) consuming it occasionally (more than once a month); and 35.4% (79/223) rarely (once in a month). The most preferred pork meal cooking method was frying 68.2% (152/223). Boiling before frying was preferred by 28.7% (64/251), boiling before roasting 1.3% (3/223) while exclusive boiling and roasting each had 0.9% (2/223). A small proportion 1.6% (4/223) slaughtered pigs at home. Government meat inspectors conduct the inspection at slaughterhouses, while on home slaughter residents do not seek the services of meat inspectors. Majority of the respondents purchase pork from butcheries 85.3% (214/251). A total of 75.3% (189/251) of respondents deworm their pigs to control worms. Worms in pigs were controlled by administration of oral anthelminthic either in tablet or powder formulation dispensed in water. The most commonly used dewormers include levamisole (Wormicid^®^ tablets, Levamisole Hydrochloride 7.5%, Cosmos, Nairobi, Kenya) and piperazine (Ascarex^®^, Piperazine tartrate, Cosmos, Nairobi, Kenya). The majority of respondents 80.9% (203/251) take dewormers, among them 64% (130/203) after 3 months, 32.5% (66/203), after 6 months and 3.5% (7/203) once in year.

### Prevalence of porcine cysticercosis

**Figure 4:**
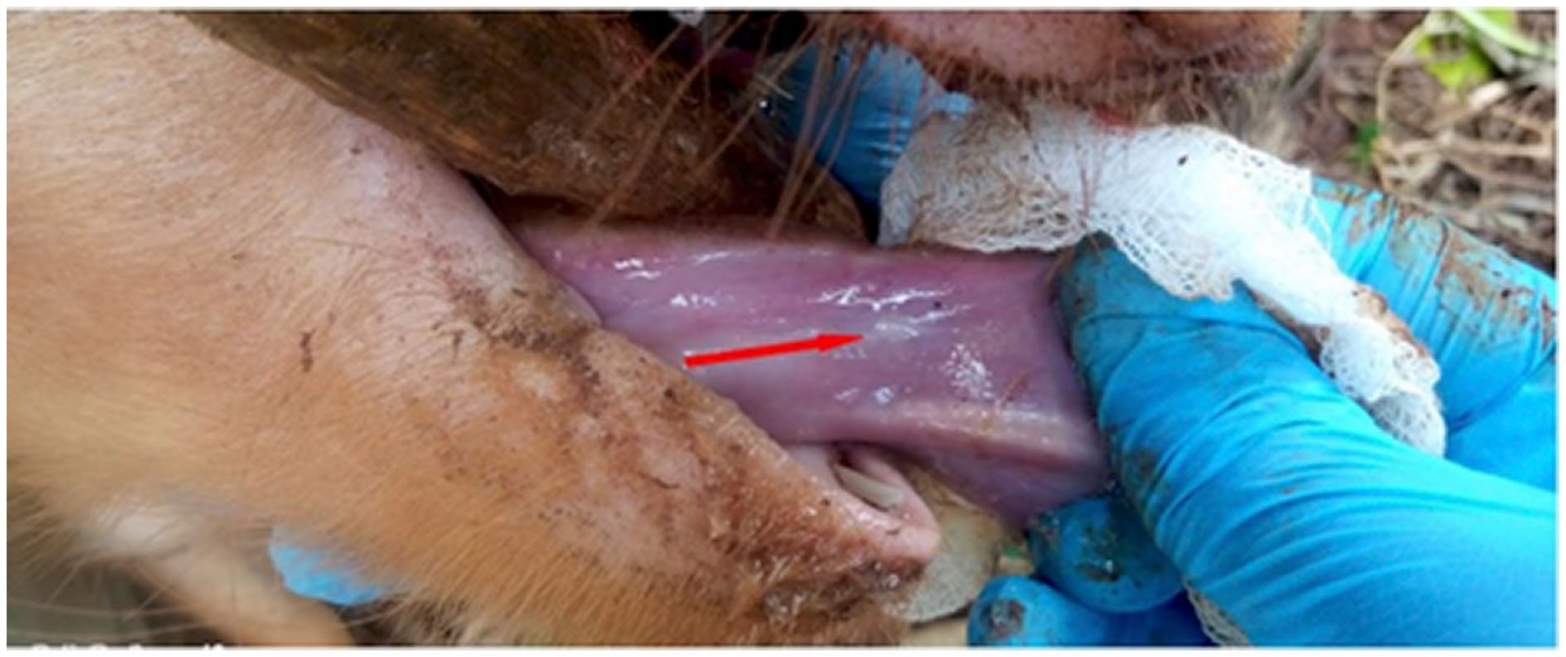
Palpable and visible cysts on the ventral side of pig tongue (red arrow).

**Figure 5:**
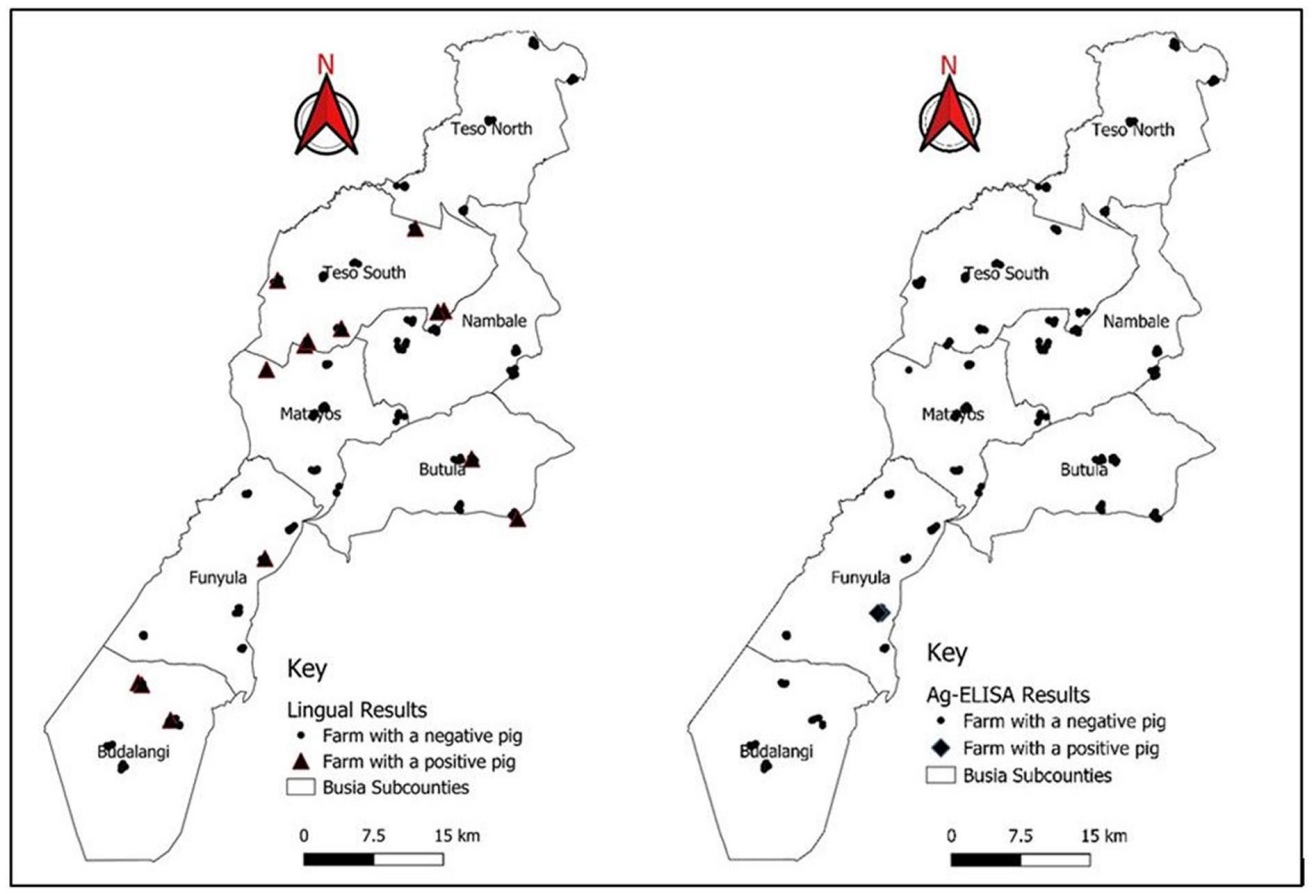
Distribution of pig keeping households in Busia County indicating the location of positive pigs by lingual palpation and apDia cysticercosis antigen ELISA.

Prevalence of porcine cysticercosis by lingual palpation during NTSP pre-implementation period in 2012 was 9.7% (9/93) (95% CI: 4.5–17.6%) while prevalence during post-implementation of NTSP was 3.8% (14/370) (95% CI: 2.3–6.3%). Figure 4 shows the tongue of a pig with a visible and palpable cyst on the ventral side. Pigs with palpable lingual cysts were detected in four out of seven sub-counties namely; Teso South (13%; 7/54), Bunyala (9.7%; 3/31) Butula (7.4%; 2/27) and Samia (4.8%; 2/42) (Table 3; Figure 5). Household prevalence by lingual palpation was 5.6% (95% CI: 3.1, 9.2%).

**Table 3:** Distribution of positive pigs on lingual palpation by Sub-county, Busia County, Kenya.

Subcounty	Number of households	Number of households with pigs positive on lingual palpation	Proportion of households with pigs positive on lingual palpation (95% CI)	
Matayos	38	0	0	0.0
Nambale	29	0	0	0.0
Teso North	30	0	0	0.0
Samia	42	2	4.8	0.6–16.2
Butula	27	2	7.4	0.9–24.1
Bunyala	31	3	9.7	2.0–25.8
Teso South	54	7	13	5.4–24.9
Totals	251	14	5.6	3.1–9.2

Prevalence of porcine cysticercosis by HP10 Ag-ELISA during NTSP pre-implementation period was 17.2% (95% CI: 10.2–26.4%), adjusted to 2.9% (95% CI: 0.1–9.6%) to account for the sensitivity and specificity of the test. The prevalence of porcine cysticercosis during post-implementation of NTSP was 0.54% (95% CI: 0.15, 1.95%), adjusted to 0.4% (95% CI: 0–1.3%). We found a statistically significant difference in the prevalence of porcine cysticercosis in Busia County between the two periods under observation, for both lingual palpation (*χ*^2^ = 5.45, *p* = 0.0196) and Ag-ELISA (*χ*^2^ = 3.73, *p* = 0.05) (Table 2).

### Risk factor analysis for the association with cysticercosis

Since the presence of palpable cysts on lingual examination of pigs is considered a possible indicator of porcine cysticercosis, we determined both pig characteristics and risk factors associated with the presence of palpable cysts on lingual examination. Univariable analysis identified that the presence of a latrine in the homestead was associated with reduced prevalence of lingual cysts in pigs (OR = 0.13; 95% CI: 0.04–0.41) and confining or tethering pigs was also associated with reduced lingual cysts (OR = 0.25; 95% CI: 0.07–0.95) (Table 4).

**Table 4:** Univariable analysis; factors associated with a positive lingual test, Busia County, 2021.

Risk factor	Level	Lingual palpation		Prevalence	Odds ratio	*p*-value	Positive	Negative	(%)	(OR)
Breed	Indigenous	10	242	4.0	1.17 (0.34–4.05)	0.80	Cross-breed/exotic	4	114	3.4		
Sex	Female	9	193	4.5	1.51 (0.49–4.70)	0.47	Male	5	163	3.0		
Age groups	Adult	3	160	1.8	0.29 (0.07–1.10)	0.07*	Young	11	196	5.3		
Latrine availability	Yes	7	208	3.3	0.13 (0.04–0.41)	<0.001*	No	7	29	19.4		
Eating pork meal	Yes	13	210	5.8	1.38 (0.17–11.51)	0.77	No	1	27	3.6		
Pigs confined or tethered all the time	Yes	3	185	1.6	0.25 (0.07–0.95)	0.04*	No	11	171	6.0		
Tapeworm shedding history	Yes	6	96	5.9	1.13 (0.36–3.54)	0.83	No	8	141	5.4		
Dewormed humans every 6 months	Yes	8	286	2.7	0.32 (0.10–1.00)	0.05*	No	6	70	7.9		
Epilepsy history	Yes	2	43	4.4	0.88 (0.18–4.30)	0.88	No	12	194	5.8		

*The final model was built using variables with *p*-value < 0.1.

The multivariable model included latrine availability, confining/tethering pigs, deworming humans every 6 months and pig age. After backward stepwise selection the final multivariable model included: latrine availability (aOR = 0.14, 95% CI: 0.05–0.43) which was significantly associated with reduced prevalence of lingual cysts in pigs and confining or tethering pigs (aOR = 0.27, 95% CI: 0.07–1.02) which was positively associated with a reduced prevalence of lingual cysts in pigs although this was not significant (Table 5). The final model had the lowest Akaike Information Criterion (AIC) of 111.2. Simulated residuals showed no obvious clustering patterns nor over dispersion, zero-inflation and outliers tests were not significant (*p-*value > 0.05).

**Table 5:** Multivariable analysis; factors associated with a positive lingual test, Busia County, 2021.

Term	Odds ratio	C.I.	*p*-value
Latrine available (yes)	0.14	0.05–0.43	<0.001
Confined or tethered	0.27	0.07–1.02	0.053

## Discussion

This study demonstrated a significant reduction in the prevalence of porcine cysticercosis in Busia County using both lingual palpation and Ag-ELISA tests after the implementation of an external, third party total sanitation program. The apparent prevalence of porcine cysticercosis by lingual palpation post-NTSP was 3.8% in 2021 compared to the prevalence of 9.7% before implementation of NTSP from 2010 to 2012 (20). The average life span of pigs in this region is 5–10 months (33) so this finding represents the pig population in Busia County in 2021 since there would have been substantial turnover of pigs since the previous study. The prevalence of porcine cysticercosis in our study was also lower than that reported in other studies in Western Kenya. Prevalence in Teso district by lingual palpation was reported to be 6.5% in 2006 (34) while in Homabay County prevalence was 5.6% in 2010 (11). A study on indigenous pig management practices in rural villages of Western Kenya found a pig prevalence by lingual palpation of 4.5% from 2006 to 2008 (14).

Comparing the adjusted prevalence estimates before and after the NTSP implementation accounting for the different diagnostic tests showed a reduction in prevalence of circulating antigens by Ag-ELISA in pigs from 2.9% in 2010 to 2012 to 0.4% in 2021. The current prevalence of 0.4% is the lowest among the recent studies conducted in Kenya. Previous studies in Kenya observed prevalence estimates which are higher than the current study with reports of 32.8% in 2010 and 17.2% between 2010 and 2012 using the Ag-ELISA (11, 35). Another study on the prevalence of porcine cysticercosis among scavenging pigs in western Kenya reported a lower prevalence at 3.8% using the Ag-ELISA in 2019 (36). This combined with our results may suggest a decreasing trend in prevalence over the past decade.

The current study observed a significant improvement in latrine coverage with pit latrines being the most common sanitation facilities from 75% in 2012 to 86% in 2021. In addition, 77.8% of those who did not have toilet facilities in their compound disposed of their excreta in the neighbor’s latrine resulting in 97% utilization of toilet facilities. A survey of sanitation and hygiene in Busia County observed similar findings to the results of our study on latrine coverage and linked it to reduced cases of diarrhea (17). Improvement in sanitation in Busia County is likely to have been as a result of the NTSP campaign introduced by the Ministry of Health. The other aspect of sanitation that had improved was lockable latrine facilities. Although pig scavenging behaviour around the latrines has increased, well-built latrines fitted with lockable doors limit access to free-roaming pigs and this combined with safe disposal of excreta through proper use of the pit latrine reduces the risk of sustaining *T. solium* lifecycle in an endemic area (37). A study conducted in Tanzania showed an increased risk of porcine cysticercosis associated with an open latrine compared to a closed latrine (38).

The design of our study could not enable us to confirm a causal relationship between improved sanitation and reduction in porcine cysticercosis prevalence. However, we were able to demonstrate through multivariable modeling a relationship between the presence of latrine and the reduced prevalence of palpable cysts in pigs (aOR 0.14, 95% CI: 0.04–0.43). Previous studies observed a similar association between the presence of a latrine in the household and the prevalence of porcine cysticercosis (11, 34, 38). However, since pigs have been documented to scavenge outside their household limits (39), latrine coverage needs to be improved at the community level as demonstrated during the NTSP. We also demonstrated that confining or tethering pigs was associated with reduced prevalence of lingual cysts (aOR = 0.27, 95% CI: 0.07–1.02). This is consistent with previous studies and promotes the need to confine pigs and prevent scavenging (40). This study investigated risk factors associated with porcine cysticercosis based on results of lingual palpation since the presence of palpable cysts on lingual examination of pigs is considered an indicator of porcine cysticercosis (34) and a rapid epidemiological tool for estimating prevalence (41). We could not stratify Ag-ELISA results for analysis since only two pigs tested positive for circulating *T. solium* antigens.

Smallholder pig farming is an important source of livelihood for the majority of households in Busia County. The results of this study portray the characteristics of a typical pig keeper in this environment which may be important when developing future control measures. The majority of pig farmers interviewed in this study were women. This is consistent with other reports that pig management in East Africa is predominantly the role of women (42, 43).

The majority of pig farmers were smallholders keeping 3 pigs per homestead similar to other studies in East Africa (14, 42, 44). Pig herd sizes were small with breeding sows and piglets being the most dominant age groups. Generally, breeding boars would require additional costs of rearing making them uneconomical to keep (43). Instead of keeping boars for breeding, farmers let sows roam freely to increase their chances of mating with free-ranging boars from other farms (14, 42).

The adult pigs were predominantly reared by letting them loose to scavenge, being tethered only when the farms had been cultivated, while piglets were always free-roaming. This is consistent with findings of a study in Busia and Kakamega counties that reported the majority of farmers tethering pigs during planting and growing season while reduced tethering was during crop harvesting seasons (14). In many pig farming systems, feeding contributes heavily to the cost of production (45). A study on the spatial ecology of free-ranging domestic pigs in western Kenya found that pigs spend much of their time scavenging outside their homesteads, suggesting that these pigs may be exposed to infectious agents including the infective stage of porcine cysticercosis (39). Considering only 5.6% of pigs are reared under total confinement with the rest tethered or free-roaming dependent on seasons, pigs may access *T. solium* eggs in the environment.

Water is a vital natural resource in any community contributing to improved hygiene, social-economic development and poverty eradication. The study showed that wells were the main water source for residents of Busia County. This contradicts the findings of a baseline survey of sanitation and hygiene in Busia County which observed that the main source of drinking water was borehole (17). Another study in western Kenya reported the use of well water being positively correlated with the presence of antigens in humans including cysticercosis, indicating that contamination of well water with fecal pollutants is common (9, 19). This is because wells are shallow, most of them are not well covered and floodwater might find its way into the well.

Knowledge of porcine cysticercosis was low with less than 1% of participants aware of the transmission routes. Future interventions may target education of pig keepers to improve knowledge of this public health hazard. A study on the effect of health education in reducing porcine cysticercosis in Mbulu District, Tanzania reported a considerable reduction in the incidence of porcine cysticercosis as measured by antigen-ELISA in sentinel pigs (46, 47).

Limitations of this study include the lack of a reliable diagnostic test that could be used as a gold standard to obtain a true prevalence since carcass dissection (48) was not practically feasible because of the cost and time involved. The lingual examination as a diagnostic test has low sensitivity which could potentially result in underestimating the prevalence of porcine cysticercosis. Since only two pigs testing positive on a serological test, further work needs to be done to ascertain if *T. solium* is circulating using a more appropriate method such as fine carcass dissection. It should also be noted that it was not possible to measure the impact of the first cross-sectional study from 2010 to 2012 on the awareness of porcine cysticercosis and improved pig keeping practices and quantify a relationship with reduced porcine cysticercosis prevalence.

## Conclusion

There was a significant reduction in the prevalence of porcine cysticercosis in Busia County across the two time periods. The low prevalence of antibodies to *T. solium* obtained in this study is a potential indicator of the reduced risk of exposure of pigs to *T. solium* eggs in Busia County. We found a significant improvement in latrine coverage and use facilitating proper disposal of human feces and reducing accessibility by free-roaming pigs which may have led to the observed reduction in the prevalence of porcine cysticercosis.

### Recommendations

There is a need to enhance intervention strategies to eliminate *T. solium* cysticercosis through improved sanitary infrastructure and pig husbandry practices in rural endemic areas. An integrated control strategy will help to break the transmission cycle of the parasite in both pigs and humans. The findings of this study indicate that implementation of community-led total sanitation (CLTS) strategy and integrating it with other control measures could successfully disrupt the transmission of porcine cysticercosis bringing it under control. This may be more rigorously tested using a randomized control trial.

Approaches that should be considered for control programs include: community health education campaigns to sensitize pig farmers, butchers, traders on porcine cysticercosis control; deworming programs for treatment of human *T. solium* and other soil-transmitted helminths; improve and sustain sanitation by construction and maintenance of latrines; and enforcement of laws, policies and guidelines at local levels on pig management. There is a need for collaboration between medical and veterinary services under a One Health approach for surveillance and control of this disease.

Future research work should be undertaken in the following areas: determination of the prevalence and economic impact of taeniosis and epileptic cases reported amongst residents of Busia County; and validation of tests used in the diagnosis of porcine cysticercosis including serological assays, meat inspection and lingual palpation.

## Data availability statement

The original contributions presented in the study are included in the article/supplementary material, further inquiries can be directed to the corresponding author.

## Ethics statement

The animal study was reviewed and approved by International Livestock Research Institute Institutional Animal Care and Use Committee. Written informed consent was obtained from the owners for the participation of their animals in this study.

## Author contributions

BC and EC contributed to conception and design of the study, performed the statistical analysis, and revised the manuscript. BC and GN conducted data collection. BC and AK analysed the data. MO, EF, and EC supervised the research. BC wrote the first draft of the manuscript. All authors contributed to the article and approved the submitted version.

## Funding

This work was part-funded by the Global Challenges Research Fund (GCRF) One Health Regional Network for the Horn of Africa (HORN) Project, from UK Research and Innovation (UKRI) and Biotechnology and Biological Sciences Research Council (BBSRC) (project number BB/P027954/1). This study received support from the CGIAR One Health Initiative “Protecting Human Health Through a One Health Approach,” which was supported by contributors of the CGIAR Trust Fund (https://www.cgiar.org/funders/). The funders had no role in the decision to publish or the preparation of this manuscript. Open access publication fees are supported by the University of Liverpool institutional access fund.

## Conflict of interest

EC declared that they were an editorial board member for Frontiers at the time of submission. This had no impact on the peer review process and the final decision.The remaining authors declare that the research was conducted in the absence of any commercial or financial relationships that could be construed as a potential conflict of interest.

## Publisher’s note

All claims expressed in this article are solely those of the authors and do not necessarily represent those of their affiliated organizations, or those of the publisher, the editors and the reviewers. Any product that may be evaluated in this article, or claim that may be made by its manufacturer, is not guaranteed or endorsed by the publisher.

## Footnotes

^1^ http://CRAN.R-project.org/
